# Evaluation of the impact of a school gardening intervention on children’s fruit and vegetable intake: a randomised controlled trial

**DOI:** 10.1186/s12966-014-0099-7

**Published:** 2014-08-16

**Authors:** Meaghan S Christian, Charlotte EL Evans, Camilla Nykjaer, Neil Hancock, Janet E Cade

**Affiliations:** Nutritional Epidemiology Group, School of Food Science and Nutrition, University of Leeds, Leeds, LS2 9JT UK; School of Health and Wellbeing, Faculty of Health and Social Sciences, Leeds Metropolitan University, City Campus, Calverley Street, Leeds, LS1 3HE UK

## Abstract

**Background:**

Current academic literature suggests that school gardening programmes can provide an interactive environment with the potential to change children’s fruit and vegetable intake. This is the first cluster randomised controlled trial (RCT) designed to evaluate whether a school gardening programme can have an effect on children’s fruit and vegetable intake.

**Methods:**

The trial included children from 23 schools; these schools were randomised into two groups, one to receive the Royal Horticultural Society (RHS)-led intervention and the other to receive the less involved Teacher-led intervention. A 24-hour food diary (CADET) was used to collect baseline and follow-up dietary intake 18 months apart. Questionnaires were also administered to evaluate the intervention implementation.

**Results:**

A total of 641 children completed the trial with a mean age of 8.1 years (95% CI: 8.0, 8.4). The unadjusted results from multilevel regression analysis revealed that for combined daily fruit and vegetable intake the Teacher-led group had a higher daily mean change of 8 g (95% CI: −19, 36) compared to the RHS-led group -32 g (95% CI: −60, −3). However, after adjusting for possible confounders this difference was not significant (intervention effect: −40 g, 95% CI: −88, 1; p = 0.06). The adjusted analysis of process measures identified that if schools improved their gardening score by 3 levels (a measure of school gardening involvement - the scale has 6 levels from 0 ‘no garden’ to 5 ‘community involvement’), irrespective of group allocation, children had, on average, a daily increase of 81 g of fruit and vegetable intake (95% CI: 0, 163; p = 0.05) compared to schools that had no change in gardening score.

**Conclusions:**

This study is the first cluster randomised controlled trial designed to evaluate a school gardening intervention. The results have found very little evidence to support the claims that school gardening alone can improve children’s daily fruit and vegetable intake. However, when a gardening intervention is implemented at a high level within the school it may improve children’s daily fruit and vegetable intake by a portion. Improving children’s fruit and vegetable intake remains a challenging task.

**Trial registration:**

ISRCTN11396528

**Electronic supplementary material:**

The online version of this article (doi:10.1186/s12966-014-0099-7) contains supplementary material, which is available to authorized users.

## Background

Epidemiological evidence indicates that a diet rich in fruit and vegetables can decrease the risk of developing cardiovascular disease, stroke, hypertension, type 2 diabetes mellitus, obesity and several forms of cancer [1–5]. A diet low in fruit and vegetable intake has been identified as one of the top 10 risk factors for global mortality [6]. Research has also revealed that dietary habits are developed in childhood and persist throughout life; therefore it is vital that children at a young age consume adequate levels of fruit and vegetables [7]. Currently, children’s consumption of fruit and vegetables is low in the United States, Australia and many European countries [8–10]. The average daily intake of fruit and vegetables for children in the UK is around 2.8 servings per day – approximately 224 g [11]- considerably lower than the recommended five portions per day. Many British children consume high levels of energy dense foods including chips, biscuits and crisps/potato chips [12]; the need for public health interventions to improve children’s overall dietary habits is therefore evident [11]. Children from low income families in the UK consume even less fruit and vegetables than the average, with boys consuming only 64 g or 0.8 of a portion and girls consuming 1.1 portions or 88 g per day [13].

Current academic literature shows promising results suggesting school gardening programmes provide an interactive environment that has the potential to change children’s self-efficacy and willingness to try different fruit and vegetables [14,15]. It is estimated that children consume approximately 20% of their dietary intake at school and school based fruit and vegetable interventions have been shown to have a moderate effect on changing children’s dietary habits [16]. School based health promotion interventions also provide an opportunity to reduce inequalities in health as all children aged 5 to 17 years are required to attend school in many countries [17]. Improvements in attitudes towards fruit and vegetables may potentially lead to an increase in actual consumption in the future. Whilst there are no published randomised controlled trials conducted on school gardening, there are gardening intervention studies that explore children’s fruit and vegetable intake using standard dietary assessment methodologies and some type of control group [15,18–21]. Parmer et al. [18] collated lunchtime observations to measure vegetable intake only, McAleese & Rankin [15], Morgan [19] and Lineberger & Zajicek [22], used 24-hour recalls to measure either fruit, vegetables or combined fruit and vegetables consumption, and Wang [20] used a three day food diary. The results from these five studies were mixed, with three [15,18,20] showing a significant difference for fruit and vegetable intake. Whereas one [22] found a difference in boys’ consumption of fruit and vegetables compared to girls’ fruit and vegetables consumption, and one [19] found no differences in fruit or vegetable intake (measured separately only). Of the studies that did show an effect on fruit and vegetable intake, two used self-selection to determine which school received the intervention [18,20]. The teacher’s willingness to teach the intervention and own beliefs in the importance of gardening could have introduced bias into these results. McAlesse and Rankin’s [15] study showed the greatest change in children’s fruit and vegetable intake, with an increase of 2.5 portions, in the garden and nutritional education group. However, the dietary tool used was administrated by the teachers and completed by the children who might be inclined to give socially desirable answers, leading to overestimation of the intervention effect. An important geographical component to acknowledge when evaluating the success of a gardening intervention is that all of the apparently successful interventions were located in states in the USA apart from Morgan [19] which was conducted in Australian regions, where fruit and vegetables can be grown all year round.

The research to date is limited by small samples with poor study designs, lack of intervention evaluations, and lack of adequate follow-up time. With the variability in quality of study design and validated tools to measure children’s nutritional intake, further research is needed to determine the potential impact gardening interventions have on children’s diets [18–20,23–25]. This study used a cluster randomised controlled trial methodology to explore how two different school gardening interventions affect children’s fruit and vegetable consumption. The aims of the study were to evaluate the impact of a school gardening programme, the Royal Horticultural Society’s (RHS) Campaign for School Gardening, on children’s fruit and vegetable intake, and to identify process measures relating to the delivery of the intervention which may affect results.

## Methods

### Study population

All primary schools (n = 1861) from the following London boroughs: Wandsworth; Tower Hamlets; Greenwich and Sutton were invited to take part in this trial, regardless of their level of previous gardening involvement in their school. Twenty-three schools responded which were then randomised. Ten were randomly allocated to receive the Royal Horticultural Society (RHS)-led and 13 schools were allocated to receive the Teacher-led intervention. The schools were randomised stratified by geographical location (London borough) using Stata [26]. All schools were allocated at the same time. No more than ten schools could receive the RHS-led intervention due to the more intensive nature of the intervention and RHS staff constraints. It was not possible in this case to randomise schools to receive no intervention at all (control/comparison group) as it is the policy of the RHS gardening charity to provide support to all schools who register an interest in their School Gardening Campaign. As a consequence of this, the second set of schools were recruited into a linked trial, Trial 2, to provide a no intervention arm - comparison group [27]. A detailed description of this study can be found in the study protocol published elsewhere [28]. Ethics approval for both trials was granted by the Leeds Institute of Health Sciences and the Leeds Institute of Genetics, Health and Therapeutic (LIHS/LIGHT) Joint Ethics Committee on 10^th^ of December 2009 (ref number HSLT/09/012).

### Sample size

The proposed sample size for this study to have 90% power to detect a 0.5 portion difference in vegetable intake per day was 627 per group, i.e. about 13 schools using 2 classes from each school. To have 90% power to detect a 1 portion difference in fruit intake per day, 482 per group was required, i.e. about 10 schools per group. The size of effect the study is powered to detect, (one half of a portion of vegetables or one portion of fruit) was chosen because it was considered the smallest improvement in intake that was worthwhile detecting with the achievable sample size, and considering the nature of the intervention. The intraclass correlation coefficient used was based on previous research with the CADET diary of 12.5% for vegetables and 11.4% for fruit intake [29].

### The intervention: the RHS campaign for school gardening

The Royal Horticultural Society (RHS) is the UK’s leading gardening charity dedicated to advancing horticulture and promoting good gardening practice. The RHS Campaign for School Gardening consists of two programmes; the Teacher-led intervention and the RHS-led intervention. The psychological theory behind school garden programmes is based on the social cognition theory (SCT). The SCT is based on the assumption that to change a person’s behaviour you need to change their knowledge, values and beliefs to be successful [30]. It is believed that active engagement in gardening activities can reinforce healthy messages about eating, and increase children’s willingness to try different fruit and vegetables. Devine [31] found that planting, growing and eating vegetables can improve children’s consumption patterns. However, there is now a gap between the implementation of school garden programmes and the academic evaluation of effectiveness [32]. The two arms of the trial are described below.

### The RHS-led intervention

The RHS-led intervention schools received the following:

- A day visit from the RHS regional advisor each half term (6 weeks) for 4 terms to work in the garden with teachers and children (Summer Term 2010 to Summer Term 2011 inclusive)
- Follow up visits to aid lead teachers with planning (Autumn Term 2011 to Autumn Term 2012)
- General ongoing advice on the school garden, free seeds and tools
- 1 twilight teacher training session each term (Summer term 2010 to Summer term 2011 inclusive), based on seasonal tasks in the school garden (open to RHS-led school teachers and others from local schools)
- Free access to a wide range of teacher resources at http://www.rhs.org.uk/schoolgardening/

The role of the regional advisor was to assist the schools to develop a successful garden, through working directly with teachers/pupils to give them support and practical advice. They were also expected to help schools overcome barriers to developing gardening within schools, with the aim of providing consistent support to each school in the intervention. The regional advisors have the expertise and experience to tie in gardening and growing activities with the National Curriculum and to run staff training sessions for teachers.

### The Teacher-led intervention

The Teacher-led intervention schools worked with the RHS by attending termly twilight training at their nearby RHS-led school, to help support them in developing and using their school garden. The regional advisor ran these twilight sessions for them and provided the Teacher-led schools with advice as needed for their school garden.

### Dietary assessment

Diet was assessed using a modified version of the validated Child And Diet Evaluation Tool (CADET) questionnaire [33]. The CADET uses age and gender specific food portion sizes to calculate daily food and nutrient intake. The CADET diary comprises a list of 115 separate food and drink types divided into 15 categories. The CADET diary for this study was split into a School Food Diary and a Home Food Diary. To complete the School and Home Food diaries, participants ticked each item consumed, under the appropriate meal time heading within the 24-hour period. The School Food Diary was completed by trained fieldworkers (who were nutrition students blinded to intervention allocation) at school for all school time meals through observing the children at each meal event. The children were given the Home Food Diary to take home for their parents to complete. A DVD with instructions for completing the questionnaire was sent home for parents/carers and children to watch (http://www.youtube.com/watch?v=AIbzqaJiHq0). The fieldworker checked the Home Food Diary when returned by the child the next day and was able to complete any missing entries using a recall approach with the child.

### Process measures

Process evaluations are used to improve the understanding of successful or unsuccessful health interventions; to identify the key components that make an intervention successful, for boys, girls or both; and to determine which environments/conditions lead to these particular components facilitating a successful outcome [34,35]. The RHS has already had a substantial external review exploring recruitment of schools, quality of the intervention materials, children’s appreciation of the intervention and learning outcomes achieved [36]. Therefore, for this trial we aimed to explore new aspects of delivery (dose received), and implementation of the various types of gardening being undertaken in the schools.

### School gardening level interview

The Gardening questionnaire was designed to identify the level of implementation and involvement of the schools in the different interventions. The school gardening level is a measurement developed by the RHS to evaluate each schools involvement in gardening based on the following scale [37].

- Zero: No garden
- Level 1: Planning
- Level 2: Getting Started
- Level 3: Growing and Diversifying
- Level 4: Sharing Best Practice
- Level 5: Celebrating with the Wider Community

To move from one level to the next the school needs to demonstrate more involvement in school gardening, in terms of development, teaching and interacting in the wider community. At baseline, each school completed a telephone interview to assess their gardening level. This interview was completed again at follow-up to assess change in gardening level.

### Gardening process measures questionnaires

The main aim of the process evaluation was to capture details about the gardening activity within each school. A gardening process measures questionnaire was designed to identify the different gardening activities that were occurring in each school and which year groups were involved. This information was captured via email in September 2010 and again at follow-up in December 2011. Some of the primary questions were:

- Which year groups are involved in gardening at your school?
- What fruit and vegetables has your school grown/tried to grow this summer?
- What did you harvest?
- What were your success/failure stories in the school garden this summer?

### Statistical analysis

#### Baseline characteristics

School level baseline characteristics were compared between the two groups. This was done to confirm that randomisation resulted in broadly similar groups in terms of weights of foods, nutrients, individual and school level characteristics. Balance of school, class and child-level variables between the two intervention groups was assessed using the following variables: school and class level - percentage of children with English as an additional language, percentage of non-white children and percentage of children with free school meal eligibility; and at child level - gender and age [24].

Statistical analysis was carried out using Stata IC version 11 [26]. The analysis was performed using clustered multilevel regression models with mean change in total daily fruit and vegetable intake as the primary outcome. Multilevel models take into consideration the hierarchical structure of the data, caused by randomising by cluster such as by school rather than by individual [38]. The multilevel regression model was used to explore the mean change in fruit and vegetable intake (follow-up intake minus baseline) between the two intervention groups. This methodology of change in fruit and vegetable intake was used rather than adjusting for baseline to meet the regression assumptions. Using intention to treat analysis methodology the models were first conducted unadjusted, and then adjusted for age, gender, ethnicity and Index of Multiple Deprivation Score (IMDS). These potential confounders were included in the models *a priori* based on a path analysis diagram created to explore factors which could assist or prevent the success of the intervention on the primary outcome [27]. The output generated for the primary analysis was effect size, standard error, 95 percent confidence intervals and p-values, with a p-value of less than 0.05 taken to represent statistical significance for all of the analyses. The same statistical methodology was applied to explore how the implementation of the intervention affected children’s fruit and vegetable intake, however this analysis was conducted ignoring intervention allocation. Comparisons were made between school and home consumption of fruit and vegetables in both groups.

## Results

### Sample size

#### Study population

**Figure 1:**
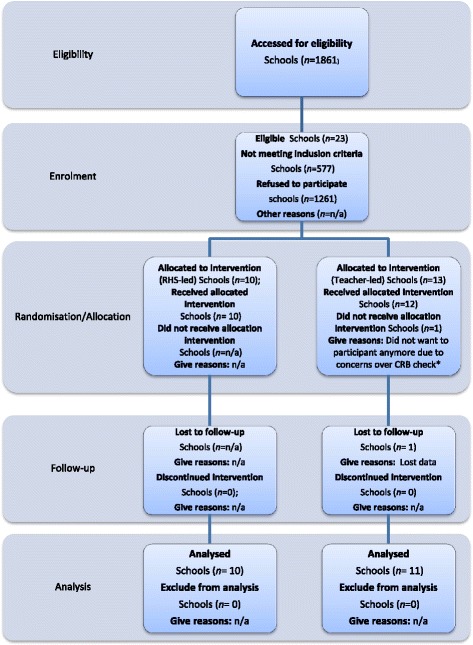
**Trial 1 RHS Gardening CONSORT Flowchart of schools.** Legend: *CRB: Criminal Records Bureau check.

**Figure 2:**
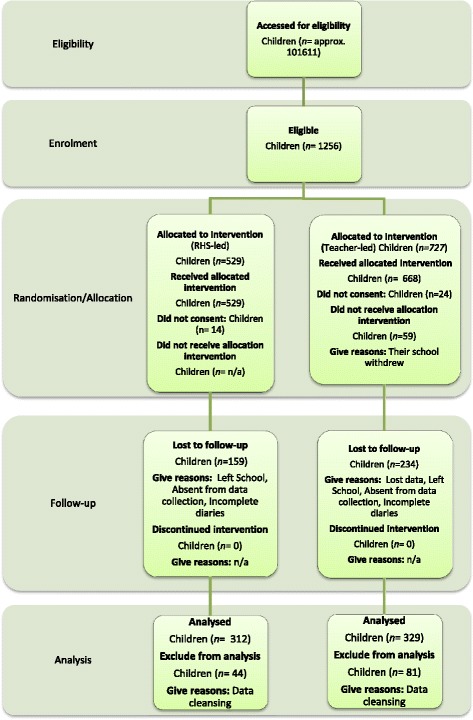
**Trial 1 RHS Gardening CONSORT Flowchart of children.**

All non-fee paying primary schools within the following London boroughs: Wandsworth; Tower Hamlets; Greenwich; and Sutton with classes in key stage 2 (years 3–6, aged 7 to 11 years) were invited to take part in the study, whether or not they had a school garden already. Independent schools fee paying, special schools and schools without all 4 year groups in key stage 2 at primary school (years 3–6) and small schools with fewer than 15 pupils per year group were excluded. Of the 1256 children who started the trial, 641 children in total completed all aspects as illustrated in Figures 1 and 2 (RHS-led: 312, Teacher-led: 329). This gave a response rate of 51 percent at follow-up. Since we estimated that 964 children were required to complete the trial, the lower response rate reduced the power to detect a difference of 0.5 portions of fruits or vegetables per day between groups from 90 to 83 percent. Of the 10 schools allocated to receive the RHS-led intervention all 10 schools completed follow up with a total of 312 children. Of the 13 schools allocated to receive the Teacher-led intervention 11 schools completed follow up with a total of 329 children. Of the schools who had dropped out, one school changed head teacher before the intervention could be delivered and withdrew from the study; and the other school posted their follow up data back to Leeds which was lost in transit despite the use of registered mail.

#### General descriptives

Table 1 describes the demographic details for the children who completed the trial. The children’s age and percentage of boys and girls and ethnicity were balanced by group. There was a substantial difference in free school meal percentage, with the RHS-led group having 33% of children receiving a free school meal compared to 24% in the teacher-led group.

**Table 1:** **Demographic information for children by intervention allocation**

	**RHS-led (n=312)**		**Teacher-led (n=329)**			
	**Mean**	**SE**	**95% CI**	**Mean**	**SE**	**95% CI**
**Child characteristic**						
Age (years)	8.2	0.07	8.1, 8.4	8.1	0.06	8.0, 8.3
Boys (%)	50			51		
**Ethnicity n (%)**						
White	92 (30)			117 (35)		
Mixed	18 (6)			22 (7)		
Asian or British Asian	72 (23)			39 (12)		
Black or British Black	38 (12)			55 (17)		
Chinese or other ethnic group	10 (3)			8 (2)		
Prefer not to say	82 (26)			88 (27)		
**School characteristic**						
FSME (%)	33			24		
IMD score	34	0.89	32.2, 35.8	30	0.78	27.9, 31.0
Children with English as an additional language (%)	54			38		

FSME: Free school meal eligibility;

IMD score: Index of multiple deprivation score.

Table 2 shows the baseline nutrient and food intake for all the children who completed baseline and follow-up. Mean energy intake per day was very similar between groups; for the (RHS-led: 2034 kcal, 95% CI: 1956, 2111; Teacher-led: 1993 kcal, 95% CI: 1925, 2059). There was a small difference in fruit and vegetable intake, with the Teacher-led group consuming on average more vegetables (RHS-led mean: 87 g, 95% CI: 78, 95; Teacher-led mean: 102 g, 95% CI: 93, 110) and more total fruit (RHS-led mean: 201 g, 95% CI: 183, 219; Teacher-led mean: 214 g, 95% CI: 195, 232). The baseline nutrient and food intakes are generally similar in terms of levels of nutrients; which suggest there was no substantial imbalance between the two groups.

**Table 2:** **Baseline nutrient intake for all children who completed baseline and follow-up collection (per day)**

	**RHS-led (n = 312)**			**Teacher-led (n = 329)**		
	**Mean**	**SE**	**95% CI**	**Mean**	**SE**	**95% CI**
Energy (kcal)	2034	39.4	1956, 2111	1993	34.1	1925, 2059
Energy (KJ)	8552	164.9	8227, 8876	8375	143.0	8103, 8666
Protein (g)	75	1.8	71, 78	73	1.5	69, 75
Carbohydrate (g)	265	4.4	256, 273	267	4.3	259, 275
Fibre (Englyst) (g)	13	0.3	12, 13	13	0.3	12, 13
Fat (g)	82	2.3	77, 86	78	1.7	74, 81
Total sugars (g)	132	2.9	126, 137	134	2.6	128, 138
Iron (mg)	11	0.2	10, 11	11	0.2	10, 11
Calcium (mg)	861	21.6	818, 903	858	18.7	821, 895
Potassium (mg)	2771	54.7	2663, 2878	2784	51.3	2683, 2884
Sodium (mg)	2632	76.3	2481, 2782	2572	57.6	2458, 2685
Folate (μg)	227	5.3	216, 237	224	4.5	214, 232
Carotene (μg)	1956	98.8	1765, 2146	2352	101.7	2152, 2552
Vitamin A (retinol equiv) (μg)	400	25.1	350, 449	403	22.7	358, 448
Vitamin C (mg)	108	3.7	100, 115	105	3.5	98, 112
Total vegetables (non-pulses, bean, lentil, dahl or seed) (g)	87	4.4	78, 95	102	4.3	93, 110
Pulses, beans, seeds (g)	16	2.2	12, 20	21	2.4	16, 25
Total fruit (g)	201	9.3	183, 219	214	9.5	195, 232
Fruit (non-dried) (g)	201	9.1	182, 218	211	9.5	191, 229
Dried fruit (g)	3	0.6	1, 3	3	0.7	2, 4
Total fruit & vegetables (excluding pulses & beans) (g)	269	10.7	248, 290	300	10.5	278, 320
Sweets, toffees, mints (g)	5	0.7	3, 6	4	0.5	2, 4
Chocolate bars, Mars etc. (g)	9	1.0	6, 10	7	0.9	5, 9
Crisps, savoury snacks (g)	12	1.0	10, 14	10	0.8	8, 11
Milk or milky drink (ml)	138	8.9	120, 153	106	7.6	91, 120
Fizzy pop, squash, fruit drink (ml)	163	11.4	141, 185	163	11.8	139, 185
Fruit juice (pure) (ml)	119	8.5	102, 135	112	7.6	95, 126

#### Change in daily fruit and vegetable intake

Table 3 displays the change in fruit, vegetables, combined fruit and vegetables (follow-up minus baseline), as well as broken down to explore fruit and vegetables intake at school and at home. Table 3 also displays the intervention mean difference, unadjusted and adjusted for IMD score, age, gender and ethnicity. The intraclass correlation in the unadjusted model was 0.003, therefore 0.3% of the variation was at the school level for change in total fruit and vegetable intake. For both groups there was a small but statistically non-significant decrease in fruit intake after adjusting for possible confounders (RHS-led: −8 g, 95% CI: −69, 52; Teacher-led: −20 g, 95% CI: −36, 77). For vegetable consumption there were also no significant differences found for either the unadjusted or adjusted model (intervention effect: −13 g, 95% CI: 39, 11). The Teacher-led group did have on average, a higher mean change in vegetable consumption, of 29 g (95% CI: −6, 66) compared to 16 g (95% CI: −11, 38) in the RHS-led group; this difference was not statistically significant. For combined fruit and vegetable intake there was a borderline significant difference in the unadjusted model (intervention effect: −40 g, 95% CI: −80, 1; P = 0.05) with the Teacher-led group having a small increase, mean change of 8 g (95% CI: −19, 36) and the RHS-led group consuming less, mean change of -32 g (95% CI: −60, −3). However, after adjusting for possible confounders this difference was not significant (intervention effect: −40 g, 95% CI: −88, 1; p = 0.06). The change in fruit and vegetable consumption at school and at home was also explored, however no differences were found between the Teacher-led group and the RHS-led group for either fruit, vegetables or combined fruit and vegetable intake at school only or at home only (Table 3).

**Table 3:** **Intervention effect on change in fruit and vegetables (g/day) between baseline and follow up**

**Food**	**RHS-led (n = 312)**			**Teacher-led intervention (n = 329)**			**Intervention effect**			
	**Mean (g)**	**SE**	**95% CI**	**Mean (g)**	**SE**	**95% CI**	**Mean diff (g)**	**SE**	**95% CI**	**P- value**
**Unadjusted**										
Change in fruit (g)	−33	11.8	−56, −10	−6	11.5	−28, 16	−27	16.4	−59, 6	0.1
Change in vegetables (g)	2	9.0	−15, 20	16	8.6	−1, 32	−13	12.4	−38, 11	0.3
Change in combined fruit and vegetables (g)	−32	14.5	−60, −3	8	14.0	−19, 36	−40	20.2	−80, 1	0.05
Change in fruit (g) School only	−36	12.9	−63, −9	−21	9.1	−40, −3	−15	15.8	−48, 18	0.3
Change in fruit (g) Home only	1	7.7	−15, 17	18	11.4	−5, 42	−17	13.8	−45, 11	0.2
Change in vegetables (g) School only	−11	7.4	−26, 4	−2	6.4	−15, 12	−9	9.9	−29, 11	0.3
Change in vegetables (g) Home only	7	4.8	−3, 17	18	6.3	5, 31	−11	8.0	−27, 5	0.1
Change in combined fruit & vegetable consumption (g) School only	−47	16.3	−81, 13	−22	8.6	−40, 4	−25	18.4	−63, 13	0.2
Change in combined fruit & vegetable consumption (g) Home only	8	9.4	−11, 28	34	16	0, 68	26	18.7	−12, 65	0.1
**Adjusted for IMDS** ^**a**^ **, Ethnicity, Age & Gender**										
Change in fruit (g)	−8	30.8	−69, 52	−20	29.0	−36, 77	−28	16.4	−60, 3	0.08
Change in vegetables (g)	16	19.6	−11, 38	29	18.2	−6, 66	−13	12.8	−39, 11	0.2
Change in combined fruit and vegetables (g)	1	39.4	−75, 78	41	36.7	−27, 116	−40	22.8	−88, 1	0.06
Change in fruit (g) School only	−25	10.1	−46, −5	−12	14.2	−41, 17	−13	13.4	−41, 14	0.3
Change in fruit (g) Home only	−32	15.8	−65, 0	−13	14.1	−42, 16	−19	14.8	−50, 40	0.2
Change in vegetables (g) School only	−8	9.6	−28, 12	−1	5.9	−12, 12	−7	9.6	−26, 12	0.4
Change in vegetables (g) Home only	12	11.2	−11, 36	23	11.0	1, 46	−11	8.7	−29, 7	0.2
Change in combined fruit & vegetable consumption (g) School only	−25	10.8	−48, −3	−4	16.0	−37, 29	−21	15.8	−54, 11	0.2
Change in combined fruit & vegetable consumption (g) Home only	−19	20.8	−62, 24	9	20.4	−33, 52	−28	20.7	−71, 14	0.2

Multi-level robust cluster regression analysis used to test significant difference between the two groups.

^a^IMDS: index of multiple deprivation score.

### Differences in nutrient and key foods

Overall, there was very little difference between the intervention groups for nutrient and food intakes (see Table 4). The mean differences were small for nearly all nutrients and foods except for energy and carotene intake. Whilst there were differences in mean intakes for both, these were not statistically significant. The only significant difference was for vitamin C. Once the adjustments were made there was a 13 mg per day (95% CI 2, 23) difference between the RHS-led and Teacher-led groups, with the Teacher-led group having a significantly higher intake of vitamin C.

**Table 4:** **Intervention effect on essential nutrient intake at follow up**

**Food**	**RHS-led (n = 312)**			**Teacher-led Intervention (n = 329)**			**Intervention effect**			
	**Mean (g)**	**SE**	**95% CI**	**Mean (g)**	**SE**	**95% CI**	**Mean diff (g)**	**SE**	**95% CI**	**P-value**
**Unadjusted**										
Total energy intake (KJ/day)	7266	524.6	6237, 8294	7388	506.4	6396, 8381	−122	435.3	−730, 976	0.8
Total energy intake (kcal/day)	1729	124.8	1485, 1974	1757	120.4	1521, 1994	−28	103.8	−175, 231	0.8
Total fat intake (g/day)	75	5.4	64, 85	73	5.1	63, 83	2	5.5	−13, 9	0.7
Sodium (mg/day)	2426	179.2	2075, 2777	2394	170.7	2060, 2729	31	188.7	−401, 338	0.9
Total sugars (g/day) including non- milk extrinsic sugars	87	6.7	74, 100	96	6.7	83, 109	−9	5.2	−1, 19	0.08
Carotene intake (mg/day)	1788	189.4	1416, 2159	1967	188.4	1598, 2337	179	236.2	−283, 642	0.4
Vitamin C intake (mg/day)	74	6.1	62, 86	87	5.9	76, 99	13	5.7	2, 24	0.2
Iron (μg/day)	9	0.7	8, 11	9	0.7	8, 11	−0.3	0.3	−0.9, 1.4	0.8
Fibre (g/day)	11	0.9	1, 13	12	0.9	11, 14	0.2	0.8	−0.0, 0.3	0.2
Carbohydrates (g/day)	213	15.4	183, 244	219	15.3	189, 249	−5.5	10.8	−15, 26	0.6
Folate (μg/day)	180	12.5	155, 204	189	12.1	166, 213	−9.9	10.9	−11, 3	0.4
Protein (g/day)	64	4.7	55, 73	69	4.5	60, 78	−5.2	4.7	−14, 4	0.3
**Adjusted for age, IMDS** ^**a**^ **, ethnicity & gender**										
Total energy intake (KJ/day)	6387	748.9	4920, 7855	6587	707.9	5199, 7974	−199	430.4	−1043, 644	0.6
Total energy intake (kcal/day)	1520	178.2	1171, 1870	1567	168.4	1237, 1897	−46	102.5	−247, 154	0.6
Total fat intake (g/day)	65	8.2	49, 81	64	7.7	49, 79	1	5.2	−9, 11	0.8
Sodium (mg/day)	2272	286	1711, 2833	2257	267.7	1732, 2781	16	190.4	−357, 388	0.9
Total sugars (g/day) including non- milk extrinsic sugars	90	10.5	70, 111	99	10.0	80, 118	−8	5.1	**−**18, 2	0.1
Carotene intake (mg/day)	1995	864	242, 3748	2164	878	442, 3886	168	230	−281, 618	0.5
Vitamin C intake (mg/day)	113	31.7	51, 175	125	31	64, 187	13	5.5	2, 23	0.02
Iron (μg/day)	8	1.0	6, 10	8	0.9	6, 10	−0.4	0.6	−1, 0.9	0.5
Fibre (g/day)	10	1.3	7, 13	11	1.3	9, 14	−1	0.8	−3, 1	0.1
Carbohydrates (g/day)	186	21.5	144, 228	193	20.6	153, 234	−7	10.9	−28, 14	0.5
Folate (μg/day)	169	19.7	131, 208	180	18.6	144, 217	−11	10.9	−32, 10	0.3
Protein (g/day)	58	7.1	44, 72	64	6.7	51, 77	−6	4.8	−15, 3	0.2

Multi-level robust cluster regression analysis used to test significant difference between the two groups.

^a^IMDS: index of multiple deprivation score.

#### Process measures results: implementation of gardening activities in schools

A detailed description of the changes made from baseline to follow-up for the RHS-led school gardens are described in the Additional file 1: Table S1 (online only). All ten of the RHS-led schools attended at least one twilight session with a mean of 3.5 (Standard Error: 0.3, 95% CI: 2.8, 4.2) sessions attended. For schools which received the Teacher-led intervention, only four schools out of the 12 attended any of the twilight sessions, with a mean of 1.5 attended (Standard Error: 0.3, 95% CI: 0.6, 2.4). At six months, four schools stated that they did not have a school garden (one from the RHS-led intervention group and three from the Teacher-led intervention group). This was reduced to two schools in the Teacher-led group and none in the RHS-led group by the end of the intervention period (see Table 5). The mean number of fruits grown at six months was 1.0 (95% CI: 0.3, 1.9) in the RHS-led group and 1.8 (95% CI: 1.6, 2.0) in the Teacher-led group, with very little change from 6 months to follow-up (RHS-led mean: 1.0, 95% CI: 0.1, 1.9; Teacher-led mean: 1.6, 95% CI: 1.4, 1.8). For mean number of vegetables grown there was very little difference at 6 months between the two groups (RHS-led mean: 6.8, 95% CI: 6.5, 7.1; Teacher-led mean: 7.2, 95% CI: 6.9, 7.8), but at follow-up the RHS-led group had a slight increase, whereas the Teacher-led group decreased in the number of different vegetables grown (RHS-led mean: 7.1, 95% CI: 6.8, 7.5; Teacher-led mean: 5.9, 95% CI: 5.6, 6.2). The most commonly grown fruit and vegetables in the RHS-led group were carrots, onions, peas, strawberries, raspberries, tomatoes and beans. Whereas, for the Teacher-led group the most common fruit and vegetables were apples, tomatoes, lettuces, strawberries, courgettes, lettuces and beans. With the RHS-led group having a wider variety of different types of fruit and vegetables grown compared to the Teacher-led group. Schools were also asked to comment on the success of their fruit and vegetable harvest. These results showed a decrease in success rate for the RHS-led group from 6 months (RHS 6 months: 50%; follow-up: 20%), whereas, the Teacher-led group had an increase from 57 percent to 100 percent successfully harvesting fruit and vegetables. This might explain in part why the Teacher-led group had on average a higher change in combined fruit and vegetable intake compared to the RHS-led group.

**Table 5:** **School gardening characteristics from 6 months to follow-up**

**Process measures**	**6 months**						**Follow-up**					
	**Teacher-led**			**RHS-led**			**Teacher-led**			**RHS-led**		
	**N**	**Mean (SE)**	**95% CI**	**N**	**Mean (SE)**	**95% CI**	**N**	**Mean (SE)**	**95% CI**	**N**	**Mean (SE)**	**95% CI**
Do you have a school garden?												
(% no)	3	25		1	10		2	17		0	0	
(% yes)	9	75		9	90		10	83		10	100	
Number of different fruits grown	9	1.8 (0.1)	1.6, 2.0	8	1.0 (0.5)	0.3, 1.9	10	1.6 (0.1)	1.4, 1.8	10	1.0 (0.1)	0.1, 1.9
Number of different vegetables grown	9	7.3 (0.2)	6.9, 7.8	8	6.8 (0.3)	6.5, 7.1	10	5.9 (0.1)	5.6, 6.2	10	7.1 (0.1)	6.8, 7.5
Size of garden (%)												
Small	1	11		1	11		0	0		0	0	
Medium	2	22		2	22		2	22		0	0	
Large	6	66		6	66		7	77		10	100	
Which year groups are involved (%)												
Reception- year 2	0	0		1	11		0	0		1	10	
Year 3- year 6	1	13		0	0		2	20		1	10	
All	7	87		8	88		8	80		8	80	
Are year 3 & year 4 involved (% yes)	7	77		8	88		9	100		9	90	
Do you have a gardening club (% yes)	6	66		6	75		6	60		7	70	
Which year groups are involved in gardening club (%)												
Reception- year 2	0	0		0	0		0	0		0	0	
Year 3- year 6	1	25		3	50		1	25		4	66	
All	3	75		3	50		3	75		2	33	
Successfully harvested fruit & vegetables (%)												
None	1	14		0	0		0	0		2	20	
Some	2	28		4	50		0	0		6	60	
All	4	57		4	50		9	100		2	20	

#### School garden level

At baseline and follow-up schools completed a Gardening Questionnaire to evaluate their gardening level using the RHS gardening level with level 0 - no garden, to level 5 – high gardening involvement, ‘celebrating with the wider community’. Fifty percent of the schools at baseline only achieved a level 1 rating compared to 60% of the schools at follow-up achieving a level 3. This shows a large improvement in the quality of the garden and gardening being integrated into the curriculum. The mean gardening level at follow-up for the RHS-led group was 2.7 (95% CI: 2.4, 2.7) compared to the Teacher-led group of 1.9 (95% CI: 1.6, 2.0). There was greater movement between the levels for the RHS-Led group compared to the Teacher-Led group (a mean increase of 1.6 compared to 1.5). Multilevel regression analysis revealed that the difference between mean change in gardening rating for the RHS-led compared to the Teacher-led group was not significant (p = 0.06).

#### School gardening level and children’s fruit and vegetable intake

To explore whether change in gardening level from baseline to follow-up was associated with the primary outcome - change in fruit and vegetable consumption, multilevel analysis was conducted using change in garden level score (follow-up minus baseline). These results are presented in Table 6. The effects on children’s fruit and vegetable intake after a positive change in one, two or three levels of gardening was compared to no change in gardening level (the reference category). An increase by one level showed little change in children’s fruit and vegetable intake, whilst increasing two levels improved children’s fruit and vegetable intake by 37 g (95% CI: −19, 96) after adjusting for age, IMDS, ethnicity and gender. Change however was only statistically significant when schools improved by three levels of the RHS gardening score; children from these schools had an average increase of 81 g (95% CI: 0, 163) of fruit and vegetables.

**Table 6:** **Mean change in fruit and vegetable intake (g/day) with change in gardening level**

	**Unadjusted**				**Adjusted for IMDS** ^**a**^ **, age, ethnicity & gender**			
**Change in gardening level**	**School (pupil) N**	**Mean change (g)**	**SE**	**p-value**	**Mean change (g)**	**SE**	**95% IC**	**p-value**
No change	8 (312)	REF			REF			
Improved by 1 level	4 (132)	−4	26.3	0.8	−5	26.9	−58, 46	0.8
Improved by 2 levels	7 (148)	30	28.9	0.2	37	29.4	−19, 96	0.1
Improved by 3 levels	2 (49)	68	41.8	0.1	81	42.0	0, 163	0.05

Multi-level robust cluster regression analysis used to test significant difference between the two groups.

REF = reference category.

^a^IMDS: index of multiple deprivation score.

## Discussion

### Daily fruit and vegetable consumption

The results for the primary outcome of the trial revealed that there was little difference in children’s mean change in fruit, vegetables or combined fruit and vegetable intake between the two groups. The Teacher-led group had slightly higher mean intakes for vegetables and combined fruit and vegetables than the Royal Horticultural Society (RHS)-led group; however there was no significant intervention effect after taking into consideration adjustment for confounders. There was also no intervention effect between the Teacher-led group and the RHS-led group for fruit, vegetables or combined fruit and vegetable intake at school only or at home only. Published studies have measured the relationship between children’s fruit and vegetable intake and a gardening intervention [15,18–21] although results are inconsistent. Out of the five studies, two reported a significant difference in fruit and vegetable intake [15,20]; one study [21] found that boys had significantly higher consumption of fruit and vegetables compared to girls; one [18] reported a significant increase in vegetable consumption only and one [19] reported no differences in fruit or vegetable intake (measured separately). The quality of previous studies is variable with some using self-selection to determine which school [18,20] or which class [19] received the intervention. In this trial, the size of the gardening area or degree of existing activities were not requirements and all schools were randomly assigned to each intervention group.

### Nutrient consumption

The differences in key nutrients and foods intakes were explored to see if there was an effect of either intervention on mean intakes. Overall there was very little difference in values for key nutrients and foods. The only significant difference was for vitamin C intake although this could not be due to increases in fruit and vegetables consumption. Once the adjustments for confounders were made there was a 13 mg per day difference between the RHS-led and Teacher-led groups, with the Teacher-led group having a significantly higher intake of vitamin C. This may be due to higher fruit drink consumption although this was not tested. Two previous studies [15,31] also explored key nutrients, identifying a significant increase in dietary fiber in the gardening intervention group compared to the control group, with McAleese and Rankin [15] also reporting a significant increase in vitamins A and C. These studies had also identified improvements in fruit and vegetable consumption.

### Intervention design, elements and geographic location

The fundamental aim of the RHS school gardening programme was to introduce children to basic gardening skills such as planting, watering, weeding, and harvesting. However, previous successful gardening interventions all involved additional elements in other settings as well as the gardening activities. Three interventions included cooking [19,20,39]; two interventions included nutrition education [15,18]. Whereas, for both the RHS-led and the Teacher-led interventions in this study, gardening was only extended into additional curriculum lessons at the school’s discretion. The primary focus of the RHS teaching is to educate children in gardening. A more holistic approach with additional complementary components to gardening might be required to achieve a more pronounced change in children’s fruit and vegetable consumption. When schools integrate gardening activities throughout their curriculum it can have a positive impact on children’s fruit and vegetable intake. Schools need support such as lesson plans demonstrating how to implement nutrition and gardening into different areas within the existing curriculum. One of the additional classes for students in a previous study was an “add a veggie to lunch day” [15]. These types of activities have shown positive results in improving children’s fruit and vegetable consumption [40]. It should also be noted that all of the successful gardening interventions were implemented in countries with warmer climates than the UK - California, Minnesota, Alabama and Florida in the US and Newcastle in Australia where fruits and vegetables are grown all year round.

The interventions for this study were either run by the RHS regional advisor or teachers within each school. Of the five successful interventions in prior trials, three of them also used teachers to implement their intervention [18–20]. If the classroom teacher was passionate about gardening, then this could potentially assist with favourable implementation of the intervention and result in wide differences in success across schools. In some previous studies [15,41], the teachers not only taught the intervention but were also trained to complete the dietary assessment which could have introduced bias into the results. Only one published study [21] had an external company similar to the RHS, the Youth Farmers and Market Project, that implemented their intervention and therefore reduced the risk of bias. A systematic review of a range of school based interventions to increase fruit and vegetable intakes in primary school aged children found a moderate improvement in fruit intake but minimal impact on vegetable intake over the whole day [11]. This suggests that any improvements in intake that may occur during school time are not necessarily maintained throughout the whole day.

### Process measures evaluation

The process measures evaluations have provided some evidence to support previous research that an intense school gardening program can improve children’s fruit and vegetable intake. The Royal Horticultural Society has a benchmarking scheme (now called School Gardening Awards) which provides a list of evidence against which schools can rate their gardening activity [42]. If schools are not doing any gardening activity this would be a level 0. The award levels 1 to 5 increase in detail and complexity including aspects of the school culture and ethos; the school garden itself; using gardening in teaching and learning and including the wider school and community in gardening. The results from this study demonstrated that whilst there was overall no significant difference in the primary outcome, when gardening in schools was implemented at an intense level and school gardening engagement substantially increased (measured by an improvement of at least 3 levels), it had a positive association on children’s fruit and vegetable intake. A limitation of the trial is that the RHS scheme involved all schools who wished to participate in the scheme, regardless of existing gardening level. There was no set minimum requirement for garden size or amount of fruit or vegetables grown. Whilst this is a positive for school gardening, it led to greater variability between the schools at baseline and potentially the level of implementation of the intervention.

It is evident that barriers to implementing a school garden program do exist. School gardens require a long term commitment [32], and a supportive team involved in maintaining the garden over the summer months when the schools are closed is crucial. The intensity as well as the sustainability of the gardening intervention could also affect the success of long term change in children’s fruit and vegetable intake [11]. All of these issues make improving children’s fruit and vegetable consumption a challenging task. The main barriers for increasing children’s fruit and vegetable intake have been identified to be availability, accessibility, convenience, taste preferences, peer pressure, parental/school support and knowledge [43]. The main barrier that teachers’ cite for not implementing school based interventions, is preparation time [44]. In this study the teacher’s continual willingness to engage with the intervention and their own beliefs in the importance of the garden, as well as daily contact with the children, could explain the current findings that, whilst not significant, the Teacher-led intervention tended to have a higher increase in fruit and vegetable consumption compared to the specialist RHS-led intervention.

### Limitations and strengths

As with many RCTs evaluating changes in dietary behaviour, there were limitations to this research. One of the disadvantages of the research design was the lack of a comparison group that received no intervention. A second trial linked to this study consisting of 1475 children was conducted with schools from London boroughs adjacent to those in this trial which were randomised to receive either the Teacher-led (n = 756) or the comparison group (n = 719). In that trial the comparison group was a delayed intervention, so that the RHS did not provide any gardening advice to these schools during the course of the trial. The results revealed that the Teacher-led group consumed on average 15 g (95% CI: −36, 148) more fruit and vegetables than the comparison group, however this difference was not statistically significant [27].

One of the main limitations of previous literature in this area is study design and the use of convenience sampling [25,45,46] and therefore a strength of this study is that it is the first cluster RCT to evaluate the effectiveness of a school gardening intervention on children’s diets. However, there are a number of potential sources of bias. The majority of the schools initially contacted did not volunteer to take part in the trial, perhaps because they are offered a range of different school programmes to choose from. This selective response may have led to differences in schools that took part compared with those that did not. In addition, many of the schools in London have a high proportion of children changing schools each academic year, which would have contributed to the high dropout rate (~30%) between baseline and follow-up.

Difficulties in delivery of the intervention and a lack of consistency of delivery may have also led to problems with analysing the effectiveness of the gardening program. Although the RHS follow an established program and aim to spend half a day every six weeks in each school, sowing, growing and harvesting the same fruits and vegetables for every school involved in the program, there may be reasons why this doesn’t happen such as the plants don’t get watered or the weather was inclement. Although efforts were made to hide the intervention group from the fieldworkers the trial was not double blind. Furthermore, schools that dramatically improved their gardening engagement during the programme may be very different in other ways not assessed in this study, Schools that initially had no garden but improved substantially may be different from schools that started with a garden but also improved their gardening engagement.

The results were analysed using a robust statistical methodology, namely multilevel analysis [47], which has the benefit that the means and confidence intervals for the different foods and nutrients are more accurate. The original plan was to measure the difference in follow-up intake of fruit and vegetables, adjusting for baseline intake. However, due to the skewed distribution of the residuals for fruit and vegetable intake, a change score was calculated [48].

A further limitation was that the sample size at baseline was lower than planned. Small sample sizes reduce the power to detect a statistical difference between groups and can lead to an overestimation of the standard errors. Nevertheless, this trial is the largest trial to evaluate school gardening to date. Furthermore, the current trial involved a highly diverse population in terms of ethnicity and socio-economic groups. Interventions targeting children living in more deprived areas have the potential to reduce inequalities in health, however the results from this trial which included many children in low socio-economic groups was not more effective than programmes with a more general appeal.

The dietary data was collected using CADET; a validated 24-hour food tick list for children aged 3–11 years old [33,49]. The strength of the CADET diary is that it uses age and gender specific food portion sizes to calculate food and nutrient intake. A one-day tick list is a less burdensome [50] and effective way of gathering nutrient information from children. However, the disadvantage of using a 24-hour tick list questionnaire is that it uses pre-allocated portion sizes for each food item which are based on average weighed intakes from UK children [5]. This method may not reflect true nutrient intake in the longer term. This study attempted to improve the quality of the dietary data by providing parents and children with an instruction DVD to help explain how to complete the CADET Home Food Diary.

### Recommendations for future research

Despite the lack of evidence of a quantitative impact of school gardening on children’s dietary behaviour reported here, the literature often describes positive attributes of school gardening identified through qualitative methods. When a school garden is successfully integrated into the school environment, it can provide a link between the community and the school. The RHS believes that school gardening can provide vital links to members of the community who otherwise have little involvement with their child’s education [37]. This is supported in academic literature [13,34] but was not assessed here.

The results from this study suggest that gardening alone, delivered at a low level of intensity, will not increase children’s fruit and vegetable consumption; however, an intense gardening programme, using a holistic approach and incorporating additional related activities with parental involvement has the potential to improve children’s fruit and vegetable intake. The authors suggest that engaging, high quality gardening interventions that also incorporate additional components such as educational activities, visits to farms and cooking programmes should be introduced into schools. The benefits of gardening programmes may be far reaching and therefore not evident for many years making evaluation difficult. Further research evaluating gardening schemes should plan follow up of more than one year to also take into account problems with weather that may reduce harvesting and productivity. Programmes could also extend to community gardens in the school vicinity that parents can get involved with. This would ensure that interventions tackle individual intake, family intake, the community as well as the school environment [32]. The World Health Organisation and Food and Agriculture Organisation believe that school based interventions play a fundamental role in improving the population’s fruit and vegetable consumption [51]. However, to improve children’s fruit and vegetable intake schools need support from the food industry and government to improve access and cost of fruit and vegetables in all settings in which children spend time.

## Conclusion

This is the first large cluster randomised controlled trial designed to evaluate a school gardening intervention in a diverse population. Little evidence was found to support the claims that school gardening alone can improve children’s fruit and vegetable intake. However, gardening interventions implemented at a high level within schools have the potential to improve children’s daily fruit and vegetable intake by a portion. Improving children’s fruit and vegetable intake remains a challenging task.

### Ethics approval

Ethical approval was obtained through the Leeds Institute of Health Sciences and Leeds Institute of Genetics, Health and Therapeutic joint ethics committee (Reference number: 09/012). Participant’s parents were given informed consent, with the opportunity to “opt-out” of the study if they did not wish their child to take part.

### Trial steering committee

The chairperson was Dr Cindy Cooper (Director, Sheffield Clinical Trials Research Unit Senior Research Fellow University of Sheffield). Graeme Slate (Learning Mentor – Forster Park Primary School, London) and Deirdre Walton (RHS Regions Manager) were the independent members of the steering committee. The Trial Steering Committee also acted as the data monitoring committee.

## Additional file

Additional file 1: Table S1. Description of the RHS school gardens at baseline and follow-up.
