# Supplementary material for: Evaluation of the impact of a school gardening intervention on children’s fruit and vegetable intake: a randomised controlled trial
Source: Int J Behav Nutr Phys Act. 2014 Aug 16;11:99. doi: 10.1186/s12966-014-0099-7 (PMC4142134; doi:10.1186/s12966-014-0099-7)
Supplement: Additional file 1: Table S1. — Description of the RHS school gardens at baseline and follow-up. [file 12966_2014_99_MOESM1_ESM.docx]

**Additional file 1**

**Table S1** Description of the RHS school gardens at baseline and follow-up

| School | Baseline | Follow-up |
| --- | --- | --- |
| Greenwich | | |
| 1 | There two main areas: allotment garden (derelict). This is a fairly large area currently set to grass at one  end and also covered with landscape fabric and gravel. There are a few raised beds, and the last third of  the area is blocked off by a solid wooden fence which is due to come down upon the completion of an adjacent building project. There is a large Acer at both ends, with the one nearest the entrance providing shade for the grassed area. As well as a sensory garden. This is in a courtyard area surrounded on three  sides by high walls. It is in deep shade, and some thought should be taken as to planting | Now has raised beds (Two groups of five RHS Wisley staff undertook teambuilding days at the school and built 16 small raised beds and two large raised beds in a new garden area). |
| 2 | No specific ‘garden’ but there are planters/raised beds where growing is being carried out. | Now have a fairly large school garden consisting of raised beds and a green house. Bannockburn took part in the Hampton Court Flower Show’s Scarecrow competition, celebrating characters from Lewis Carroll’s famous books ‘Alice's Adventures in Wonderland‘ and ‘Through the Looking Glass’. Their ‘Mr Caterpillar’ gained a very respectable third prize in a field of more than 20 schools. |
| Tower Hamlets | | |
| 3 | The school currently has a wildlife and vegetable garden complete with pond. This area is due to be demolished to create new classrooms for this expanding school. | A willow tunnel has been created. A new garden was being built over the 2011 summer holidays. |
| 4 | Various ‘areas’:- An excellent wildlife garden. A thriving raised pond, a spider’s web design wild flower meadow, plum tree, climbers, outdoor classroom. KS2 Years 3, 4, 5 & 6 have their own large planter in  the playground. KS1 have four large planters. There are 81 meter long beds. These are used by Mums to grow stuff for the local co-op. | Already had growing areas, but now have a shed and greenhouse yet to be erected. A Muslim Mums Group has taken part in two informal Twilight sessions including seed sowing and pricking out seedlings. |
| 5 | There is currently no gardening. | Now have 5 raised beds for growing |
| Sutton | | |
| 6 | A compact garden consisting of attractive gravel paths, four large raised beds, and a fenced off pond (including a small deck). There are other planters and beds around the school grounds, including some  small planters in the Early Years playground planted up with herbs. | In addition to their raised beds, now has a greenhouse |
| 7 | There is one main Garden which has a number of beds and a thriving pond. A small newly cultivated  bed in a shady area is planted up with a number of suitable plants. Due to fairly small total growing area, there is limited quantity, which affects the whole school exposure. There is currently no sheltered growing area to raise plants | Now have new beds built by parents (the school held two ‘Get your Grown-ups Growing’ events over the winter, when parents took part in digging, and the construction of new beds) |
| Wandsworth | | |
| 8 | The current garden is extremely impressive, but there is little provision for the children to grow  (in terms of growing beds). The delightful garden is known as the ‘secret garden’ and has many features: a ‘Human Sundial’ in the centre, a small lawn, wildlife area with properly layered hedge, trees, a bog area, various benches, and one small vegetable area (approx. 1.5 m x 3 m). There are also some raised brick planters in the main playground, which have mainly permanent planting and herbs. These beds are rather | Now have two new growing areas. Development on the school grounds is ongoing. |
| 9 | There are a number of areas set out for growing: Main Garden comprised of 8 raised beds/planters  (four of which are thin planters approx. 40 cm) A polycarbonate greenhouse has been purchased and is  to be built next to the Nursery garden. There are 3 raised beds in a separate courtyard area which year 1 use. | In addition to their 8 raised beds, the school now has two large growing beds built by parents (the school held a ‘Get your Grown-ups Growing’ event over the winter) and a greenhouse. |
| 10 | A few small raised beds in the main school garden which have been neglected somewhat. The timber is starting to break as the beds are made of a number of compost bin kits. There is no fence around the  garden which allows the children to play on and in the beds. The school has acquired a large allotment  plot (1 min walk from the school). The aim is to turn this into a community garden, and use the produce for the school kitchens. This plot is totally overgrown at present. | In addition to developing their own thriving school garden, the School has taken responsibility for a plot of land on the adjacent housing estate. This is to be a School Community Garden. In conjunction with the Residents Association and with the support from the RHS, this area is gradually being developed. |
